# Semi-supervised spectral clustering with application to detect population stratification

**DOI:** 10.3389/fgene.2013.00215

**Published:** 2013-10-25

**Authors:** Binghui Liu, Xiaotong Shen, Wei Pan

**Affiliations:** ^1^Division of Biostatistics, School of Public Health, University of MinnesotaMinneapolis, MN, USA; ^2^School of Statistics, University of MinnesotaMinneapolis, MN, USA

**Keywords:** clustering, genome-wide association studies (GWAS), population stratification, semi-supervised spectral clustering, single nucleotide variant (SNV)

## Abstract

In genetic association studies, unaccounted population stratification can cause spurious associations in a discovery process of identifying disease-associated genetic markers. In such a situation, prior information is often available for some subjects' population identities. To leverage the additional information, we propose a semi-supervised clustering approach for detecting population stratification. This approach maintains the advantages of spectral clustering, while is integrated with the additional identity information, leading to sharper clustering performance. To demonstrate utility of our approach, we analyze a whole-genome sequencing dataset from the 1000 Genomes Project, consisting of the genotypes of 607 individuals sampled from three continental groups involving 10 subpopulations. This is compared against a semi-supervised spectral clustering method, in addition to a spectral clustering method, with the known subpopulation information by the Rand index and an adjusted Rand (ARand) index. The numerical results suggest that the proposed method outperforms its competitors in detecting population stratification.

## 1. Introduction

With the rapid advance of high-throughput technologies, genome-wide association studies (GWAS) and whole-exome or whole-genome sequencing studies have become popular (International HapMap Consortium, 2003). However, in a population-based association study, presence of undetected population stratification, also referred to as the population structure, becomes a potential issue leading to false discovery (Marchini et al., 2004). Population stratification occurs in presence of a systematic difference in allele frequencies between cases and controls due to different ancestries. One direct consequence of ignoring population stratification is inflated false positives and false negatives (Lander and Schork, 1994; Hirschhorn and Daly, 2005; Thomas et al., 2005).

Clustering has been an effective means to detect and describe known or cryptic population stratification (Paschou et al., 2010). For detecting or adjusting for population stratification, three major methods have been proposed, including genomic control (Devlin and Roeder, 1999; Devlin et al., 2004), structured association mapping and other clustering methods (Pritchard et al., 2000; Satten et al., 2001), and principal component analysis [PCA, (Patterson et al., 2006; Zhang et al., 2012)] and spectral methods (Lee et al., 2009; Zhang et al., 2009). As argued in Lee et al. (2009), different methods may be applicable in different situations, for instance, a combination of PCA and a clustering method may be preferable when the method is applied to preprocess in association studies. Despite progress, issues remain. One important issue is how to utilize additional prior information to enhance clustering performance to adjust for population stratification. In a situation where some subjects' population identities are known *priori*, a semi-supervised approach is more suitable. Towards this end, we propose two methods to detect population stratification, that is, semi-supervised clustering methods that are integrated with PCA and another clustering method, respectively. These methods are developed to (1) integrate the prior information for clustering, (2) to avoid that dense clusters are collapsed into a single group, whereas their sparser counterparts are divided into more multiple clusters, and (3) utilize the prior information to separate highly overlapped subpopulations.

For (1), we incorporate prior information through constraints, as in Grira et al. (2004). The constraints are expressed in terms of pairwise *must-links* and *cannot-links* imposed over a subset of the subjects with known population identities, where a must-link connects two subjects from the same subpopulation, whereas a cannot-link deals with different subpoluations.

For (2), we develop our semi-supervised clustering method based on a local scale spectral clustering method (Zelnik-manor and Perona, 2004). In some situations, subpopulations may not be in a same scale, then we consider a spectral clustering method involving a local scaling parameter to guard against potential disruptive influence caused by the different densities of the different subpoluations.

For (3), we introduce a continuous parameter to adjust similarities between subjects with cannot-links and those without any cannot-link, in addition to adjusting similarities between must-link pairs and cannot-link pairs. As indicated in the numerical results in Section 3.1, many pairs of subjects with cannot-links in two different subpoluations were assigned into one cluster by an existing semi-supervised method, which is in contrast to the proposed semi-supervised spectral clustering method.

The paper is organized as follows. Section 2 gives a motivating data example, and introduces the proposed methods. Section 3 presents our analysis of a low-coverage whole-genome sequencing data, published on the 1000 Genomes Project website. This is followed by a discussion in Section 4.

## 2. Materials and methods

### 2.1. Data

In this study, we used a low-coverage whole-genome sequencing dataset to evaluate the performance of our semi-supervised spectral clustering algorithm. The processed data were downloaded from the 1000 Genomes Project (1000 Genomes Project Consortium 2010) web site http://www.sph.umich.edu/csg/abecasis/MACH/download/1000G-2010-08.html. The pha-sed data contain the DNA sequences of *n* = 607 individuals of three continental groups: Africans (AFR), Europeans (EUR) and Asians (ASN); there are 3, 4, and 3 subgroups in the three continental groups respectively (Table 1) after we removed three subgroups (2 PUR and 1 MXL) from the downloaded data due to their small sample sizes.

**Table 1:** **10 subgroups of 607 individuals**.

**AFR:**	**YRI**	**LWK**	**ASW**	
# Samples	78	67	24	
label	1	2	3	
**EUR:**	**GBR**	**FIN**	**CEU**	**TSI**
# Samples	43	36	90	92
label	4	5	6	7
**ASN:**	**CHS**	**CHB**	**JPT**	
# Samples	25	68	84	
label	8	9	a	

We used all the *p* = 7,459,664 SNVs appearing in all the three continental groups on chromosomes 1 to 22. In the 7,456,664 SNVs, there are 343,782 rare variants (RVs, with minor allele frequencies, MAFs < 1%), 1,189,061 low frequency variants (LFVs, 1%≤ MAFs < 5%) and 5,926,821 common variants (CVs, MAFs ≥ 5%). There are 132,742, 525,440 and 1,107,080 monomorphic variants in each of the three continental groups: AFR, EUR and ASN, respectively, and there are 18,559 variants that are monomorphic in all the three continental groups. Furthermore, there are 101,279 variants that are monomorphic in AFR but polymorphic in EUR, and 67,661 variants that are monomorphic in AFR but polymorphic in ASN; there are 493,977 variants that are monomorphic in EUR but polymorphic in AFR, and 133,388 variants that are monomorphic in EUR but polymorphic in ASN; there are 1,041,999 variants that are monomorphic in ASN but polymorphic in AFR, and 715,028 variants that are monomorphic in ASN but polymorphic in EUR.

Denote the data by an *n* × *p* matrix *Z*, with rows indexing *n* individuals, and columns indexing *p* SNVs. For each SNV, we chose the minor allele as the reference allele. Let *Z*_*ij*_ ∈ {0, 1, 2} be the number of minor alleles for SNV *j* of individual *i*. We centered each column (SNV) to have mean 0; denote the centered data matrix *Z*_*c*_ = *AZ*, where $A=I-\frac{1}{n}11^{t}$ is an *n* × *n* centering matrix, *I* denotes the *n* × *n* identity matrix and **1** denotes the length-*n* vector with each entry equal to 1. Then, we used PCA for dimension reduction (Menozzi et al., 1978; Cavalli-Sforza et al., 1994): we computed the *n* × *n* sample covariance matrix *H* = *Z*_*c*_ *Z*^*t*^_*c*_, and then used the re-scaled eigenvectors of *H* as coordinates for subject *i*, $x_{i}=(\sqrt{\lambda_{1}}u_{1}(i),\ldots ,\sqrt{\lambda_{J}}u_{J}(i))$, where λ_1_ ≥ λ_2_ ≥ ··· ≥ λ_*J*_ ≥ 0 are the *M* largest eigenvalues of *H* and *u*_*j*_ = (*u*_*j*_(1), …, *u*_*j*_(*n*))^*t*^, *j* ∈ {1, …, *J*}, are the corresponding eigenvectors. Typically, eigenvectors that correspond to large eigenvalues reveal important ancestry axes.

### 2.2. Semi-supervised spectral clustering

Existing semi-supervised clustering methods can be categorized into two: search-based and similarity-based. The former is a modified clustering method in that the prior constraints are used to yield appropriate partitions (Demiriz et al., 1999; Wagstaff et al., 2001; Basu et al., 2002). The latter is a clustering method based on a modified similarity metric (Bilenko and Mooney, 2003; Xing et al., 2003; Yang et al., 2008). We think that the latter may be more efficient, since it embeds prior constraints only by simply modifying the similarity metric, while the former may use prior constraints to yield appropriate partitions in each iteration.

With this in mind, in this paper we developed a semi-supervised spectral clustering method to infer population structure. Before proposing this method in detail, we first review some spectral clustering algorithms, which were developed from the studies of weighted graph partitioning problems (Shi and Malik, 2000; Meila and Shi, 2001; Ng et al., 2001; Kannan et al., 2004). The spectral clustering algorithms are similarity-based. A popular choice for defining the similarity between a pair of subjects (*x*_*i*_, *x*_*j*_) (*i* ≠ *j*) is letting *W*_*ij*_ = exp(−||*x*_*i*_ − *x*_*j*_||^2^/σ^2^_*ij*_), where the scale parameter σ_*ij*_ controls the size of local neighborhoods in the weighted graph. Although a global scale σ_*ij*_ = σ is often used, as mentioned in Zelnik-manor and Perona (2004), using a local scale parameter, σ_*ij*_ = (σ_*i*_σ_*j*_)^1/2^ with σ_*i*_, σ_*j*_ > 0, for each pair (*i, j*) may obtain better performance, especially when the clusters of the data have different volumes. Below we review the local scale spectral clustering algorithm proposed by Zelnik-manor and Perona (2004).

Given a set of *n* points {*x*_1_, …, *x*_*n*_} in the *J*-dimensional Euclidean space **R**^*J*^ and the neighborhood parameter *T*, cluster them into *K* clusters as follows:

1. Compute alocal scale σ_*i*_ = *d*(*x*_*i*_, *x*_*i*_*T*__) for each point *x*_*i*_, where *d*(.,.) is the Euclidean distance metric and *x*_*i*_*T*__ is the *T*-th nearest neighbor of point *x*_*i*_.
2. Form a weight matrix *W* with its *ij*-th element $W_{ij}=\exp(-\frac{d^{2}(x_{i},x_{j})}{\sigma_{i}\sigma_{j}})$ for each *i* and *j* ∈ {1, …, *n*} with *i* ≠ *j* and *W*_*ii*_ = 0 for each *i* ∈ {1, …, *n*}.
3. Define *D* to be a diagonal matrix with *D*_*ii*_ = ∑^*n*^_*j* = 1_ *W*_*ij*_ and construct the normalized Laplacian matrix 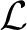 = *I* − *D*^−1/2^ *WD*^−1/2^.
4. Find *u*_1_, …, *u*_*K*_, the smallest *K* eigenvectors of 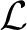, and let *U* be the matrix containing the vectors *u*_1_, …, *u*_*K*_ as columns.
5. For *i* = 1, …, *n* and *j* = 1, …, *K*, let *U*′_*ij*_ = *U*_*ij*_/(∑^*K*^_*j*′ = 1_*U*^2^_*ij*′_)^1/2^, and then *U*′ is a matrix with elements *U*′_*ij*_.
6. For *i* = 1, …, *n*, let *y*_*i*_ = (*y*^(1)^_*i*_, …, *y*^(*K*)^_*i*_)^*t*^ ∈ **R**^*K*^ be the vector corresponding to the *i*th row of *U*′, and then cluster the points {*y*_1_, …, *y*_*n*_} with the K-means algorithm into *K* clusters {*S*_1_, …, *S*_*K*_}.
7. Assign the original point *x*_*i*_ to cluster *S*_*j*_ if and only if *y*_*i*_ is assigned to cluster *S*_*j*_.

Let 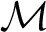 denote the must-link matrix and 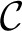 denote the cannot-link matrix for clustering *n* points {*x*_1_, …, *x*_*n*_}, where 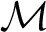
_*ii*_ = 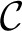
_*ii*_ = 0 (*i* = 1,…,*n*) and for *i* ≠ *j*, 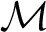
_*ij*_ = 1 (or 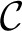
_*ij*_ = 1) means that *x*_*i*_, *x*_*j*_ are already known to be in the same (or different) cluster(s); 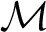
_*ij*_ = 0 (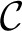
_*ij*_ = 0) means that we do not know whether *x*_*i*_, *x*_*j*_ are in the same cluster. Given the must-link matrix 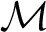 and the cannot-link matrix 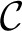, Yang et al. (2008) proposed a semi-supervised algorithm by modifying the second step of the local scale spectral clustering algorithm above as follows: for each pair *i* ≠ *j*, if 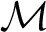
_*ij*_ = 1, then let *W*_*ij*_ ≡ 1; if 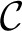
_*ij*_ = 1, then let *W*_*ij*_ ≡ 0. By letting *W*_*ij*_ ≡ 1 for a must-link pair (*i, j*), the algorithm forces the pair (*i, j*) to be clustered into the same cluster. However, in general, letting *W*_*ij*_ ≡ 0 for a cannot-link pair (*i, j*) may not force the pair (*i, j*) to be clustered into two different clusters. *W*_*ij*_ ≡ 0 only means that observations *i* and *j* are far away; if there exists an observation *k* such that *W*_*ik*_ and *W*_*kj*_ are large enough, *i* and *j* may still be clustered into one cluster. Thus, for embedding cannot-link information into a spectral clustering algorithm, only letting the weights of all cannot-link pairs to be zero is not enough. To avoid clustering a cannot-link pair (*i, j*) into one cluster, we adjust *W*_*ik*_ and *W*_*kj*_ for each *k* without any cannot-link information, based on which we propose a new semi-supervised spectral clustering algorithm.

Before introducing the algorithm, to make best use of the semi-supervised information, we may first adjust the must-link matrix and cannot-link matrix as follows: (1) Adjust the must-link matrix 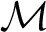 such that: for each pair (*i, j*) (*i* ≠ *j*), 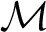
_*ij*_ = 1 whenever there exists a *k* ≠ *i, j* such that 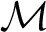
_*ik*_ = 1 and 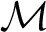
_*kj*_ = 1; (2) Adjust the cannot-link matrix 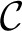 such that: for each pair (*i, j*) (*i* ≠ *j*), 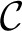
_*ij*_ = 1 whenever there exists a *k* ≠ *i, j* such that 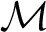
_*ik*_ = 1 and 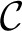
_*kj*_ = 1. After the adjustment, if there exists any contradictory pair (*i, j*) (*i* ≠ *j*) with 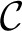
_*ij*_ = 1 and 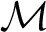
_*ik*_ = 1, to avoid being misled we will let 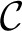
_*ij*_ = 0 and 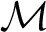
_*ik*_ = 0.

In fact, though there have been much reported success with using pairwise constraints for clustering, there are two limitations (Davidson and Ravi, 2005; Davidson et al., 2006). First, if the constraints are poorly specified and then using cannot-link constraints may make the feasibility problem intractable (Davidson and Ravi, 2005); second, some constraints may have adverse effects to semi-supervised clustering (Davidson et al., 2006). There were some discussions about how to deal with the limitations, and accordingly some methods were specifically designed to overcome such limitations. Because the concern of the limitations is not the focus of this paper, we will not introduce these methods in detail.

Let *V*_
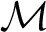
_ = ∪_
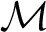
_*ij*_ = 1_{*i, j*} and *V*_
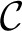
_ = ∪_
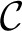
_*ij*_ = 1_{*i, j*}. Now we are ready to show our semi-supervised spectral clustering (SSSC) algorithm.

**Algorithm SSSC** Given a set of *n* points 𝒟 = {*x*_1_, …, *x*_*n*_} in **R**^*J*^, a must-link matrix 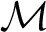, a cannot-link matrix 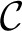, and the parameters α ∈ {0, 1}, β ≥ 1 and the neighborhood parameter *T*, cluster the points into *K* clusters as follows:

1. Compute the local scale σ_*i*_ = *d*(*x*_*i*_,*x*_*i*_*T*__) for each point *x*_*i*_, where *x*_*i*_*T*__ is the *T*-th neighbor of point *x*_*i*_.
2. Form the weight matrix *W*:
  - Initially let $W_{ij}=\exp(-\frac{d^{2}(x_{i},x_{j})}{\sigma_{i}\sigma_{j}})$ for *i* ≠ *j* and *W*_*ii*_ = 0.
  - For each *i* and *j* (*i* ≠ *j*), if 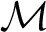
_*ij*_ = 1, let *W*_*ij*_ = 1; and if 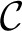
_*ij*_ = 1, let *W*_*ij*_ = 0.
  - For each *k* ∈ *V*\*V*_
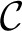
_, let *c*_*k*_ = arg max_*c*∈*V*_
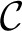
__(*W*_*kc*_). Then for each *i* ∈ *V*_
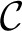
_ with 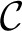
_*c*_*k*_*i*_ = 1, let *W*_*ik*_ and *W*_*ki*_ be replaced by *W*_*ik*_/β.
  - For each *k* ∈ *V*\*V*_
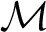
_, let *m*_*k*_ = arg max_*m*∈*V*_
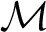
__(*W*_*km*_). If α = 1, then for each *j* ∈ *V*_
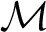
_ with 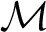
_*m*_*k*_*j*_ = 1, let *W*_*jk*_ and *W*_*kj*_ be replaced by *W*_*m*_*k*_*k*_.
3. Define *D* to be a diagonal matrix with *D*_*ii*_ = ∑_*j*_ *W*_*ij*_ and construct the normalized Laplacian matrix 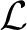 = *I* − *D*^−1/2^*WD*^−1/2^.
4. Find *u*_1_, …, *u*_*K*_, the first *K* eigenvectors of 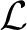, and let *U* be the matrix containing the vectors *u*_1_, …, *u*_*K*_ as columns.
5. For *i* = 1, …, *n* and *j* = 1, …, *K*, let *U*′_*ij*_ = *U*_*ij*_/(∑^*K*^_*j*′ = 1_*U*^2^_*ij*′_)^1/2^, and then *U*′ is a matrix with elements *U*′_*ij*_.
6. For *i* = 1, …, *n*, let *y*_*i*_ = (*y*^(1)^_*i*_, … *y*^(*K*)^_*i*_)^*t*^ ∈ **R**^*K*^ be the vector corresponding to the *i*th row of *U*′, and then cluster the points {*y*_1_, …, *y*_*n*_} with the K-means algorithm into *K* clusters {*S*_1_, …, *S*_*K*_}.
7. Assign the original point *x*_*i*_ to cluster *S*_*j*_ if and only if *y*_*i*_ was assigned to cluster *S*_*j*_.

Note that in the Step 2.c of our new algorithm above, we believe that for each *k* ∈ *V*\*V*_
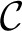
_ and each cannot-link pair (*i, j*), if *x*_*k*_ is nearer to *x*_*i*_, then it should be much farther away from *x*_*j*_, because the distance between *x*_*i*_ and *x*_*j*_ has already been set to the maximum. Thus, we penalize the similarity between *x*_*k*_ and *x*_*j*_ by letting *W*_*jk*_ = *W*_*kj*_ = *W*_*jk*_/β (β > 1). On the other hand, we set a parameter α to determine whether we force the similarities between a sample *k* ∈ *V*\*V*_
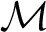
_ and a must-link pair (*i, j*) to be the same. In fact, if α = 0 and β = 1, then our algorithm reduces to that of Yang et al. (2008).

### 2.3. Choosing the parameters

We develop a cross-validation procedure to choose the parameters for the **Algorithm SSSC**, modified from a criterion used in Tibshirani and Walther (2005) for the K-mean clustering. In addition, we borrow the idea of cluster reproducibility index (RI) (Shen et al., 2009) to define a new prediction strength. We summarize the procedure as follows.

Given a data set 𝒟 and a candidate set of parameters Θ = 𝒦 × 𝒜 × ℬ × 𝒯, where 𝒦 and 𝒯 are sets of positive integers, 𝒜 = {0, 1}, and ℬ is a set of real numbers equal to or larger than 1. Randomly permute the sample index set *V* = [*N*] of 𝒟, and then partition the permuted sample index set into two roughly equal parts. Select one part as the test index subset *V*^*te*^ for the test data 𝒟^*te*^ = {*X*_*n*_: *n* ∈ *V*^*te*^} and take the remaining part as the training index subset *V*^*tr*^ for the training data 𝒟^*tr*^ = {*X*_*n*_: *n* ∈ *V*^*tr*^}. Let 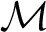
^*tr*^ = 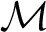
_*V*^*tr*^*V*^*tr*^_, 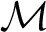
^*te*^ = 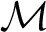
_*V*^*te*^*V*^*te*^_, 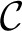
^*tr*^ = 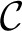
_*V*^*tr*^*V*^*tr*^_ and 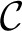
^*te*^ = 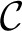
_*V*^*te*^*V*^*te*^_. For each θ = (*K*, α, β, *T*) ∈ Θ, apply **Algorithm SSSC** to divide 𝒟^*tr*^ into *K* clusters with parameters α, β, *T* and the must-link matrix 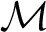
^*tr*^, the cannot-link matrix 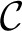
^*tr*^; apply **Algorithm SSSC** to divide 𝒟^*te*^ into *K* clusters with parameters α, β, *T* and the must-link matrix 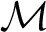
^*te*^, the cannot-link matrix 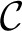
^*te*^. Let *l*_*tr*_ and *l*_*te*_ denote the corresponding clustering assignments. Divide the test data 𝒟^*te*^ into *K* clusters under the guidance of *l*_*tr*_, that is, assign each sample in 𝒟^*te*^ into the closest cluster of 𝒟^*tr*^ characterized by *l*_*tr*_ in the sense of the Euclidean distance, and then let *l*_*te|tr*_ denote the corresponding clustering assignment. Note that here the distance between a sample and a cluster is defined as the minimum distance between this sample and each sample in the cluster. Next, compute the adjusted Rand index (Hubert and Arabie, 1985) between *l*_*te|tr*_ and *l*_*te*_ as the prediction strength. Repeat the above steps for a number of times with different randomly selected permuted samples, and finally choose $\hat{\theta }=(\hat{K},\hat{\alpha },\hat{\beta },\hat{T})\in \Theta$ with the highest average prediction strength.

Note that while using PCA for dimension reduction in Section 2.1, we did not mention how to choose an appropriate number of PCs. There are many studies about this problem for traditional PCA, such as Jackson (1991), Jolliffe (2002) and Pedro et al. (2005). Because in this paper we only focus on the performance of a clustering algorithm, we propose using a special procedure that is related to the clustering performance. In fact, we view the number of PCs as a parameter and then decide it in the above cross-validation procedure. Especially, we first choose ${\hat{\theta }}_{J}\in \Theta$ using the above cross-validation procedure for each *J* in a set of candidate numbers of PCs 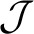, and then choose *Ĵ* ∈ 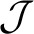 with the highest average prediction strength among 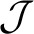 as the best fitted number of PCs.

## 3. Results

### 3.1. Main results

**Figure 1:**
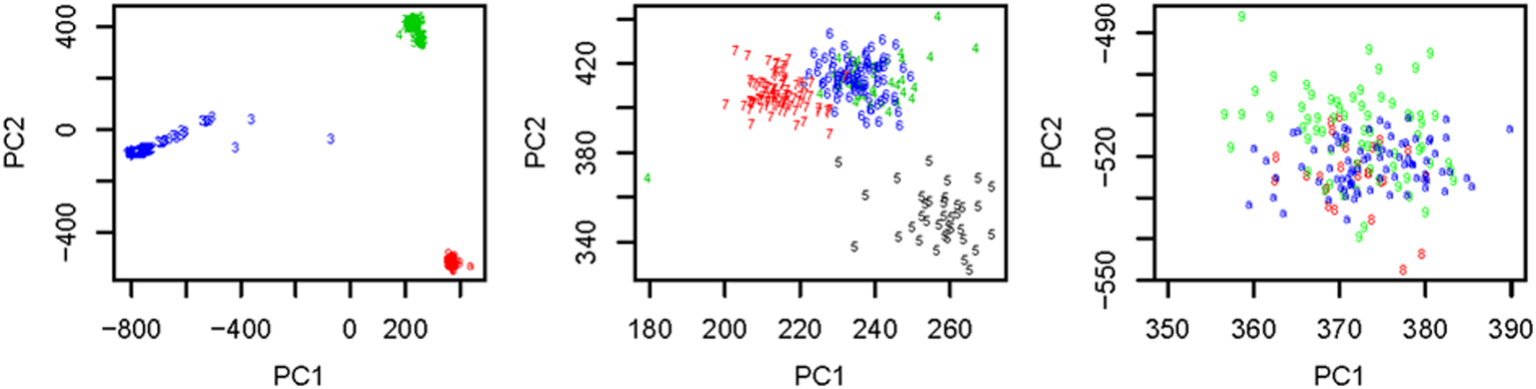
**Left:** Top: 2 PCs after PCA dimension reduction for the SNV data from chromosomes 1-22, where subjects are self-identified as EUR (green), AFR (blue) and ASN (red). **Middle:** Top 2 PCs for EUR group: GBR (green), FIN (black), CEU (blue), TSI (red); **Right:** Top 2 PCs for ASN group: CHS (red), CHB (green), JPT (blue).

We used all the SNVs appeared in all the three continental groups in chromosomes 1-22 to extract the top *t* principle components (PCs). As shown in the left panel of Figure 1, the top 2 PCs could completely separate the three continental groups. However, some subgroups could not be completely separated. We used the local scale spectral clustering algorithm introduced in Section Methods to cluster the 607 *t*-dimension vectors into 10 clusters. As shown in Table 2 and Figure 1, subgroup GBR (‘4’) cannot be completely distinguished from CEU (‘6’); CHS (‘8’) cannot be completely distinguished from CHB (‘9’).

**Table 2:** **The numbers of subjects assigned to each of the 10 clusters based on the top 10 PCs using the unsupervised local scale spectral clustering algorithm**.

**Groups**	**Subgroups**	**S1**	**S2**	**S3**	**S4**	**S5**	**S6**	**S7**	**S8**	**S9**	**Sa**	**All**
AFR	YRI	78	0	0	0	0	0	0	0	0	0	78
	LWK	0	60	7	0	0	0	0	0	0	0	67
	ASW	0	0	1	23	0	0	0	0	0	0	24
EUR	GBR	0	0	4	0	39	0	0	0	0	0	43
	FIN	0	0	0	0	0	36	0	0	0	0	36
	CEU	0	0	0	0	90	0	0	0	0	0	90
	TSI	0	0	2	0	0	0	90	0	0	0	92
ASN	CHS	0	0	0	0	0	0	0	25	0	0	25
	CHB	0	0	0	0	0	0	0	68	0	0	68
	JPT	0	0	0	0	0	0	0	0	17	67	84

(Rand = 0.961, ARand = 0.821).

**Figure 2:**
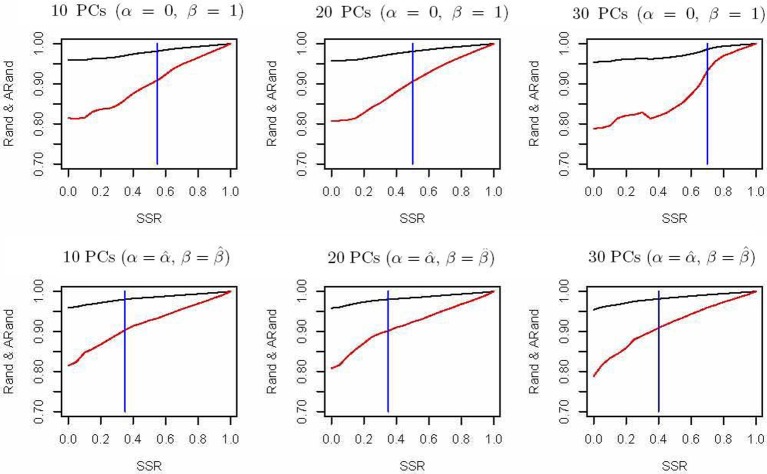
**Rand indices (black) and adjusted Rand indices (red) between the true subgroups and the clustering results with various numbers of the top PCs.** A vertical blue line indicates the point of *SSR*, at which the Rand index and adjusted Rand index are both larger than 0.90 for the first time.

The spectral clustering algorithm used above is an unsupervised clustering algorithm without using any additional clustering information. However, in many cases, partial knowledge is available concerning pairwise (must-link or cannot-link) constraints among a subset of subjects. Thus, we propose a semi-supervised local scale spectral clustering algorithm to make use of the pre-known constraints. We show the performance of our algorithm by varying the number of available must-link or cannot-link constraints. We let SSR denote the semi-supervised ratio, and randomly selected a fraction SSR of individuals from each subgroup. Then we obtained a must-link matrix and a cannot-link matrix according to the selected individuals and their subgroup identities, which were input to our semi-supervised algorithm. We used the algorithm in Yang et al. (2008) and our new proposed algorithm to cluster the 607 individuals into 10 clusters with the top 10, 20, and 30 PCs respectively, and then compared the Rand index (Rand, 1971) and an adjusted Rand (ARand) index (Hubert and Arabie, 1985) between the true subgroups and the clustering results. We repeated this process for 100 times and at each time we randomly selected some individuals for getting the pre-known must-link matrix and cannot-link matrix by setting a different seed in **R** software. Then we indicated the average results of these 100 simulations in Figure 2. From Figure 2, we can see that when using the top 10, 20 and 30 PCs for clustering, our algorithm performed much better than the existing one with (α = 0, β = 1) (Yang et al., 2008) in terms of the Rand index and adjusted Rand index for almost all the values of SSR. It is clear that the blue vertical lines for our new algorithm appeared with smaller SSR values, indicating that our algorithm made use of the given semi-supervised information more efficiently. In fact, while using other numbers of the top PCs, we also obtained similar results (not shown). Additionally, here in our new algorithm we used α = $\hat{\alpha }$ and β = $\hat{\beta }$.

Tables 3, 4 present the numbers of subjects assigned to each of the 10 clusters based on the top 10 PCs using the existing SSSC algorithm (α = 0, β = 1) (Yang et al., 2008) and our **Algorithm SSSC** with SSR = 0.5. We can see that our new algorithm performed much better than the existing one.

**Table 3:** **The numbers of subjects assigned to each of the 10 clusters based on the top 10 PCs using the existing SSSC algorithm (α = 0, β = 1) with SSR = 0.5**.

**Groups**	**Subgroups**	**S1**	**S2**	**S3**	**S4**	**S5**	**S6**	**S7**	**S8**	**S9**	**Sa**	**All**
AFR	YRI	78	0	0	0	0	0	0	0	0	0	78
	LWK	0	62	5	0	0	0	0	0	0	0	67
	ASW	0	0	1	23	0	0	0	0	0	0	24
EUR	GBR	0	0	2	0	39	2	0	0	0	0	43
	FIN	0	0	0	0	0	0	36	0	0	0	36
	CEU	0	0	0	0	17	73	0	0	0	0	90
	TSI	0	0	1	0	0	0	0	91	0	0	92
ASN	CHS	0	0	0	0	0	0	0	0	25	0	25
	CHB	0	0	0	0	0	0	0	0	68	0	68
	JPT	0	0	0	0	0	0	0	0	1	83	84

(Rand = 0.975, ARand = 0.881).

**Table 4:** **The numbers of subjects assigned to each of the 10 clusters based on the top 10 PCs using our new SSSC algorithm (α = $\hat{\alpha }$, β = $\hat{\beta }$) with SSR = 0.5**.

**Groups**	**Subgroups**	**S1**	**S2**	**S3**	**S4**	**S5**	**S6**	**S7**	**S8**	**S9**	**Sa**	**All**
AFR	YRI	78	0	0	0	0	0	0	0	0	0	78
	LWK	0	67	0	0	0	0	0	0	0	0	67
	ASW	0	0	24	0	0	0	0	0	0	0	24
EUR	GBR	0	0	0	7	34	2	0	0	0	0	43
	FIN	0	0	0	0	0	0	36	0	0	0	36
	CEU	0	0	0	82	8	0	0	0	0	0	90
	TSI	0	0	0	0	0	92	0	0	0	0	92
ASN	CHS	0	0	0	0	0	0	0	20	5	0	25
	CHB	0	0	0	0	0	0	0	4	64	0	68
	JPT	0	0	0	0	0	0	0	0	1	83	84

(Rand = 0.984, ARand = 0.923).

**Figure 3:**
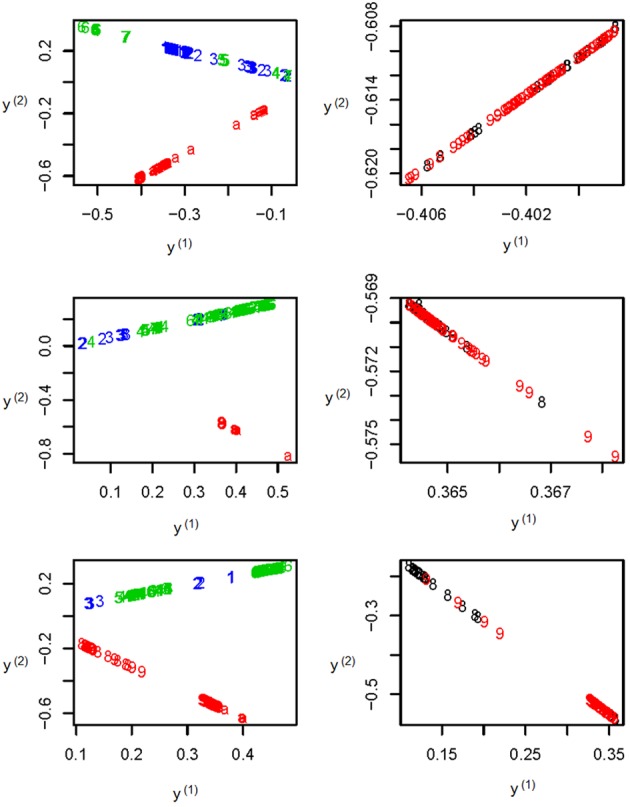
**The top two sub-figures are for the unsupervised local scale spectral clustering algorithm, the middle two are for SSSC algorithm (α = 0, β = 1) and the bottom two are for our SSSC algorithm (α = $\hat{\alpha }$, β = $\hat{\beta }$).** Note that in the right three sub-figures, the colors of the subjects in CHS (‘8’) were changed from red to black. Note that the *y*^(1)^ and *y*^(2)^ axes are just the first two co-ordinates of the data points {*y*_1_, …, *y*_*n*_} in Step 6 of **Algorithm SSSC**.

To further illustrate the difference among the unsupervised local scale spectral clustering algorithm, the existing SSSC algorithm (α = 0, β = 1) (Yang et al., 2008) and our new SSSC algorithm (α = $\hat{\alpha }$, β = $\hat{\beta }$), we plotted the first two co-ordinates (of *y*_*i*_'s in Step 6) for each of the three algorithms (see Figure 3). To better observe the separation between the two subgroups CHS (‘8’) and CHB (‘9’), we particularly plotted for the two subgroups, where the colors of the subjects in CHB were still kept red, however, those in CHS were changed to black (see the right three sub-figures of Figure 3). The top two sub-figures are for the unsupervised local scale spectral clustering algorithm, the middle two are for the existing SSSC algorithm (α = 0, β = 1) and the bottom two are for our SSSC algorithm (α = $\hat{\alpha }$, β = $\hat{\beta }$). From the first two sub-figures of Figure 3 and Table 2, we see that the two pairs of subgroups, GBR-CEU and CHS-CHB were inseparable, respectively. Then for the middle two sub-figures of Figure 3 and Table 3, by adjusting the similarities between must-link pairs to be 1 and those between cannot-link pairs to be 0, the subjects in GBR and CEU were a little more separable, however the subjects in CHS and CHB were still inseparable. Finally for the last two sub-figures of Figure 3 and Table 4, we can see that the subjects in all the subgroups were more separable, and in particular, in the bottom right sub-figure the subjects in CHS and CHB were more separable.

### 3.2. Semi-supervised clustering versus classification

We also did some numerical experiments to compare classification (supervised learning) with our semi-supervised clustering. For illustration and to have a easier problem for classification, we only took the individuals in the EUR continental group and used common variants (CVs) with minor allele frequencies (MAFs) greater than 5% on chromosome 1. Furthermore, we used PLINK (Purcell et al., 2007) to prune out correlated SNVs with a sliding window of size 50 (shifted by 5) and a threshold of *r*^2^ ≤ 0.05, after which we had 11,840 CVs.

**Figure 4:**
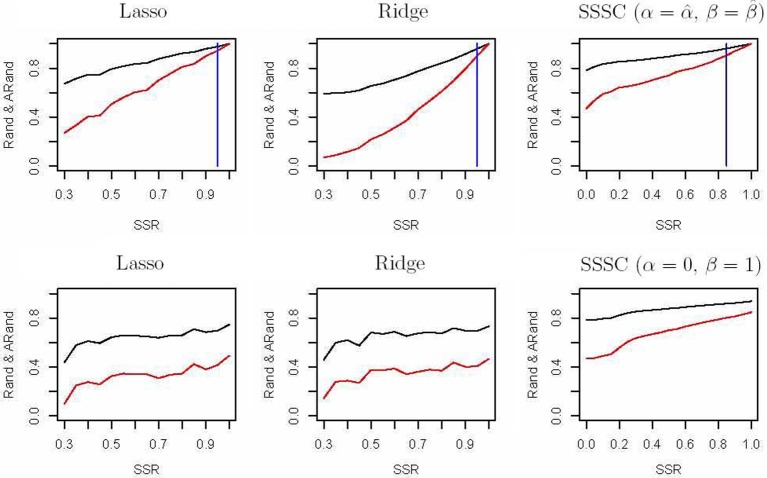
**Rand indices (black) and adjusted Rand indices (red) between the true subgroups and the SSSC or classification results.** A vertical blue line indicates the point of SSR, at which the Rand and adjusted Rand are both bigger than 0.90 for the first time. The top three sub-figures demonstrate comparison of Lasso, Ridge and SSSC for the cases that semi-supervised information involves all the four subgroups, while the bottom three for the cases that semi-supervised information involves two subgroups.

First, we randomly chose a fraction SSR of individuals from each of the above four subgroups as semi-supervised information for our clustering algorithm and as the training data for a classification algorithm. We used penalized multinomial logistic regression with the Lasso or the Ridge penalty for classification; the penalization parameter was chosen by 5-fold cross-validation. We used the trained classifier to predict the subgroup labels for the remaining data, and combined the known labels in the training data and the predicted labels in the test data together to compare with the true labels in terms of the Rand indices and adjusted Rand indices. The top two sub-figures in Figure 4 summarize the corresponding results based on 100 simulations; it is demonstrated that in our experiments, our semi-supervised clustering algorithm performed much better than both Lasso- and Ridge-penalized regression, especially for cases with low SSRs.

On the other hand, in some cases the given semi-supervised information may not involve all the four subgroups. For example, we only had information about a subset of subjects from the CEU and GBR subgroups, but not any from the other subgroups. As before, we randomly selected a fraction (*SSR*) of individuals from the CEU and GBR subgroups respectively; they were used as semi-supervised information for our algorithm and as training data for a classification algorithm. The bottom three sub-figures in Figure 4 show the corresponding comparisons, indicating that our semi-supervised clustering algorithm performed overwhelmingly better than Lasso- and Ridge-penalized regression, because the classification algorithm predicted all the individuals of the unknown TSI or FIN subgroup as of either CEU or GBR subgroup. This illustrates an obvious advantage of a semi-supervised clustering approach for discovery of novel classes.

## 4. Discussion

### 4.1. Dimension reduction

In Section 3, we have demonstrated good performance of our algorithm with a few top PCs. In addition, we also obtained similar results (not shown) with other methods for dimension reduction, such as the spectral graph approach used in SpectralR and SpectralGEM (Lee et al., 2010). We used the spectral method in (Lee et al., 2010) for dimension reduction, then clustered the data into 10 clusters; we took the same procedure to compare the Rand index and adjusted Rand index values. Figure 2.

### 4.2. All or a subset of SNVs?

In the previous section, we used all 7,459,664 SNVs appearing in all the three continental groups on chromosomes 1 to 22 without pruning out SVNs in linkage disequilibrium. We used common variants (CVs) with minor allele frequencies (MAFs) greater than 5% on chromosomes 1 to 22, and used PLINK (Purcell et al., 2007) to prune out correlated SNVs with a sliding window of size 50 (shifted by 5) and a threshold of *r*^2^ ≤ 0.5, after which we had 1,022,090 CVs. Next, we used PCA for dimension reduction and then use the three algorithms to analyze the resulting data after dimension reduction.

Tables 5–7 present the numbers of subjects assigned to each of the 10 clusters based on the top 10 PCs using the unsupervised spectral clustering algorithm, the existing semi-supervised spectral clustering algorithm and our new algorithm with *SSR* = 0.5. From these results and those indicated by Tables 2–4, we see that using all the SNVs was better than using the pruned data in terms of the performance of the unsupervised spectral clustering. For the two semi-supervised spectral clustering algorithms, we find that while using the pruned data, the new semi-supervised spectral clustering algorithm still performed better than the existing one (Yang et al., 2008) as in Section 3.1 with all the SNVs.

**Table 5:** **The numbers of subjects assigned to each of the 10 clusters based on the top 10 PCs using the unsupervised local scale spectral clustering algorithm**.

**Groups**	**Subgroups**	**S1**	**S2**	**S3**	**S4**	**S5**	**S6**	**S7**	**S8**	**S9**	**Sa**	**All**
AFR	YRI	59	19	0	0	0	0	0	0	0	0	78
	LWK	0	67	0	0	0	0	0	0	0	0	67
	ASW	24	0	0	0	0	0	0	0	0	0	24
EUR	GBR	0	0	0	43	0	0	0	0	0	0	43
	FIN	0	0	0	0	36	0	0	0	0	0	36
	CEU	0	0	0	90	0	0	0	0	0	0	90
	TSI	0	0	0	0	0	92	0	0	0	0	92
ASN	CHS	0	0	0	0	0	0	19	6	0	0	25
	CHB	0	0	0	0	0	0	21	7	40	0	68
	JPT	0	0	0	0	0	0	0	16	0	68	84

(Rand = 0.948, ARand = 0.758).

**Table 6:** **The numbers of subjects assigned to each of the 10 clusters based on the top 10 PCs using the existing SSSC algorithm (α = 0, β = 1) with SSR = 0.5**.

**Groups**	**Subgroups**	**S1**	**S2**	**S3**	**S4**	**S5**	**S6**	**S7**	**S8**	**S9**	**Sa**	**All**
AFR	YRI	78	0	0	0	0	0	0	0	0	0	78
	LWK	0	67	0	0	0	0	0	0	0	0	67
	ASW	0	0	24	0	0	0	0	0	0	0	24
EUR	GBR	0	0	0	43	0	0	0	0	0	0	43
	FIN	0	0	0	0	36	0	0	0	0	0	36
	CEU	0	0	0	12	0	78	0	0	0	0	90
	TSI	0	0	0	0	0	0	92	0	0	0	92
ASN	CHS	0	0	0	0	0	0	0	25	0	0	25
	CHB	0	0	0	0	0	0	0	10	58	0	68
	JPT	0	0	0	0	0	0	0	0	0	84	84

(Rand = 0.988, ARand = 0.941).

**Table 7:** **The numbers of subjects assigned to each of the 10 clusters based on the top 10 PCs using our new SSSC algorithm (α = $\hat{\alpha }$, β = $\hat{\beta }$) with SSR = 0.5**.

**Groups**	**Subgroups**	**S1**	**S2**	**S3**	**S4**	**S5**	**S6**	**S7**	**S8**	**S9**	**Sa**	**All**
AFR	YRI	78	0	0	0	0	0	0	0	0	0	78
	LWK	0	67	0	0	0	0	0	0	0	0	67
	ASW	0	0	24	0	0	0	0	0	0	0	24
EUR	GBR	0	0	0	43	0	0	0	0	0	0	43
	FIN	0	0	0	0	36	0	0	0	0	0	36
	CEU	0	0	0	6	0	84	0	0	0	0	90
	TSI	0	0	0	0	0	0	92	0	0	0	92
ASN	CHS	0	0	0	0	0	0	0	25	0	0	25
	CHB	0	0	0	0	0	0	0	5	62	1	68
	JPT	0	0	0	0	0	0	0	0	0	84	84

(Rand = 0.991, ARand = 0.957).

### 4.3. Local scale spectral clustering

Our semi-supervised spectral clustering algorithm is based on the local scale spectral clustering (Zelnik-manor and Perona, 2004), because we believe that local scales work better than choosing a single global scale for all pairs of subjects. In some situations the subgroups might not have the same scale; from our experience, given a fixed number of clusters, the subjects in a sparser group are more likely to be divided into more clusters, and the individuals in a denser group are more likely to be merged together. In these cases, it will be difficult to choose a suitable single global scale. In contrast, using local scales automatically adjusts for the heterogeneous scales in the subgroups. We did some experiments to compare the spectral clustering algorithms with a global scale and with local scales. We used several candidate values for a global scale, and found that even the best clustering result (in terms of the Rand indices and adjusted Rand indices) was almost the same as that obtained by using local scales. Because it is not the main point of this study, we do not show the detailed comparisons here.

## 5. Conclusions

We have proposed a new semi-supervised spectral clustering algorithm based on a more efficient use of the cannot link constraints in prior data. A whole-genome sequencing dataset from the 1000 Genomes Project was analyzed to compare the performance of our and other algorithms. In our experiments, unsupervised clustering algorithms could not completely separate some subgroups, such as the CEU-GBR and CHB-CHS subgroups; our semi-supervised spectral clustering algorithm, along with a subset of individuals with known subgroup identities, distinguished these subgroups much better. Our proposed method may be potentially useful in genetic association studies. Its extensions to other clustering (Thalamuthu et al., 2006) and dimension reduction approaches are to be studied.

### Conflict of interest statement

The authors declare that the research was conducted in the absence of any commercial or financial relationships that could be construed as a potential conflict of interest.
